# Mortality in patients treated with intravitreal bevacizumab for age-related macular degeneration

**DOI:** 10.1186/s12886-017-0586-0

**Published:** 2017-10-10

**Authors:** Joel Hanhart, Doron S. Comaneshter, Yossi Freier Dror, Shlomo Vinker

**Affiliations:** 1 Department of Ophthalmology, Shaare Zedek Medical Center, 12 Beyt Street, 91031 Jerusalem, Israel; 2 Central Headquarters, Clalit Health Services, Tel Aviv, Israel; 3 Mashav Applied Research, Jerusalem, Israel; 4 Sackler School of Medicine, Tel Aviv University, Ramat Aviv, Tel Aviv, Israel

**Keywords:** Neovascular AMD, Anti-VEGF, Bevacizumab, Safety, Mortality

## Abstract

**Background:**

The aim of this study is to analyze mortality in patients treated with bevacizumab for wet AMD.

**Methods:**

We conducted a retrospective case-control study between patients who received intravitreal injections of bevacizumab as the sole treatment for exudative AMD between September 2008 and October 2014 (*n* = 5385) and age and gender matched controls (*n* = 10,756). All individuals included in the study were reviewed for sociodemographic data and comorbidities. Survival analysis was performed using adjusted Cox regression, using relevant adjusted variables.

**Results:**

During follow-up (maximum: 73 months), 1063 (19.7%) individuals after bevacizumab died compared with 1298 (12.1%) in the control group (*P* < .001). After adjusted Cox survival regression, mortality differed significantly between the groups, Odds ratio = 1.69, (95% C.I. 1.54–1.84), *P* < .001.

**Conclusions:**

We found an increased long-term mortality in individuals with wet AMD treated with bevacizumab compared to a same age and gender group without wet AMD.

## Background

Intravitreal anti-vascular endothelial growth factor (anti-VEGF) treatment has revolutionized the management of many retinal conditions, including age-related macular degeneration (AMD). Several anti-VEGF agents are used in the treatment of neovascular AMD. Ranibizumab and aflibercept are approved as ophthalmic therapies. Bevacizumab is a full-length humanized monoclonal IgG antibody of 149 kDa that inhibits all VEGF-A isoforms [1]. Approved in 2004 by the FDA, for systemic use in the treatment of certain metastatic cancers, bevacizumab is widely used off-label as intravitreal therapy in neovascular AMD since its efficacy was described more than a decade ago [2].

Reduced systemic VEGF level was demonstrated in patients who received intravitreal anti-VEGF agents, the systemic effect was most obvious with bevacizumab. Intraocular injection of bevacizumab strongly decreases VEGF serum concentration, to the extent that 1 month after the treatment, VEGF serum level is only 23% of baseline [3]. Circulating VEGF protects vascular patency and integrity [4].

In prospective studies of bevacizumab treatment for neovascular AMD, mortality was found to be 0.81%–10.00% at 1 year [5–10] and 5.07%–5.97% at 2 years [11, 12]. A recent meta-analysis of 6 randomized controlled trials reported that approximately 25% more bevacizumab-treated than ranibizumab-treated patients experienced one or more serious non-ocular adverse events over one and 2 years. Among patients who received bevacizumab, overall mortality was 1.95% at 1 year (25/1282 patients) and 5.78% at 2 years (51/882) [13]. Another meta-analysis that comprised 1623 patients reported 1.91% mortality at 1 year [14]. However, many published studies and meta-analyses were not powered enough to accurately assess the systemic risks of anti-VEGF intravitreal injections [15].

In the public health system in Israel, patients diagnosed with neovascular AMD are offered bevacizumab as a first line agent, in accordance with the efficacy demonstrated by major studies [5, 7–9, 11].

We report the mortality of all patients treated during a 6 year period, with intravitreal bevacizumab for neovascular AMD, in the largest health maintenance organization in Israel; and compare it to the mortality of age and gender-matched individuals not-exposed to bevacizumab.

## Methods

### Data sources

This retrospective, population-based analysis accessed data from the electronic medical records of all individuals affiliated with Clalit Health Services who received intravitreal injections of bevacizumab for treatment of AMD between September 2008 and October 2014.

Clalit Health Services maintains a chronic disease registry database that includes information collected from a variety of sources: primary care physician reports, medication-use files, hospitalization records, and out-patient clinic records. The methods of registry acquisition and maintenance were described by Rennert and Peterburg [16].

For all individuals included in the analysis, we extracted information from the registry regarding the following conditions, which have been reported to be more prevalent in AMD patients and to be associated with increased mortality [17–19]: smoking, alcohol abuse, ischemic heart disease, cerebrovascular disease, congestive heart failure, liver cancer, obesity, and (unilateral/bilateral) pseudophakia.

The definitions in the Clalit database of alcohol abuse are based on the Diagnostic and Statistical Manual of Mental Disorders, version IV. Cerebrovascular disease was diagnosed following the criteria of the National Institute of Neurological Disorders [20]. The clinical data standards of the American College of Cardiology/American Heart Association Task Force were used to define congestive heart failure and ischemic heart disease [21]. A body mass index of 30 kg/m^2^ or higher defined obesity.

Additional information extracted from patients’ files included age, gender, marital status, and socioeconomic status.

The date of death was automatically communicated from the Israeli Interior Ministry via the unique national identity number. The cause of death was not recorded.

Ethics approval was obtained from the Ethics Committee of the Clalit Health Services.

### Study population

In the nationwide Clalit Health Services records, we identified patients treated by anti-VEGF for wet AMD, and excluded those for whom there was doubt regarding the indication of the treatment. Forty-seven patients were excluded because it was not possible to eliminate diabetic macular oedema as the indication for injections; 29 since high myopia could not be ruled out as the cause of choroidal neovascularisation; 18 as the reason for treatment may have been a concomitant diagnosis of retinal vein occlusion; in 4 patients, inflammatory conditions were identified as the possible etiology of choroidal neovascularisation. Patients who received other intraocular anti-VEGF agents (pegaptanib, ranibizumab, aflibercept) or systemic anti-VEGF therapy at any time were excluded from the analysis.

For each wet AMD patient treated with bevacizumab in the study group, two individuals were matched in age and gender from the members of Clalit Health Services. A matched control had the same age as the case on the date of first bevacizumab injection. Criteria for this reference group were no recorded exposure to anti-VEGF and continual membership in Clalit Health Services from September 2008 until October 2014, excepting death.

### Statistical analysis

For all ratio variables, means and standard deviations were calculated and baseline differences between the groups evaluated using a t-test. For all nominal variables, absolute frequencies and percentages were calculated and baseline differences between the groups were assessed using a Chi-square test. The socioeconomic ordinal variable baseline differences between the groups were evaluated using the Mann-Whitney test. To compare mortality over time between the groups, survival analysis was performed using adjusted Cox regression. The dependent variable was survival. The time-dependent covariate for the treatment group (bevacizumab) was the interval between the first injection to survival or death; and for the control group, the interval between the start of monitoring (date of first injection in the corresponding bevacizumab treated patient) to survival or death, all truncated at 7 years. Adjusted variables were age, smoking, alcohol abuse, hypertension, diabetes, obesity, congested heart failure, liver cancer, ischemic heart disease, and cerebrovascular accident.

Statistical analyses were conducted using the SPSS statistical software (Version 20). The criterion for accepting the research hypothesis was: Alpha (α) = .05 (one-sided). The criterion for negating the preliminary differences between the treatment and the control group was: Alpha (α) = .05 (two-sided).

## Results

A total of 5385 individuals met the criteria established for the treatment group; and 10,756 aged and gender matched individuals comprised (the control group).

Sociodemographic and clinical characteristics of the groups are shown in Table 1. Patients in the treatment group were a mean 3.5 months older than controls (81.2 vs. 80.9 years). The proportion of males was the same, 45.7% in both groups. A high prevalence of medical comorbidities was found in both groups, though higher in the bevacizumab group.

**Table 1:** Patient characteristics and outcome

	Treated with bevacizumab (*N* = 5385) N (%)	Not treated with bevacizumab (*N* = 10,756) N (%)	*P*-value
Age Start [mean ± SD]	81.17 ± 8.91	80.88 ± 8.91	.051
Male	2460 (45.7)	4916 (45.7)	.979
Married	2180 (40.5)	4637 (43.1)	< .001
Socioeconomic status^a^			<.001
High	1213 (22.6)	2910 (27.1)	
Medium	2510 (46.7)	4620 (43.1)	
Low	1652 (30.7)	3190 (29.8)	
Cataract	2353 (43.7)	3585 (35.9)	< .001
Smoking	1008 (18.7)	1555 (14.5)	< .001
Alcohol	27 (.05)	64 (.06)	.524
Hypertension	4142 (76.9)	7666 (71.3)	< .001
Diabetes mellitus	1821 (33.8)	3019 (28.1)	< .001
Obesity	1413 (26.2)	2581 (24.0)	< .001
Congestive heart failure	538 (10.0)	893 (8.3)	< .001
Liver cancer	4 (.01)	6 (.01)	.913
Ischemic heart disease	2030 (37.7)	3434 (31.9)	< .001
Cerebrovascular accident	857 (15.9)	1508 (14.0)	< .001
Mortality	1063 (19.7)	1298 (12.1)	< .001

^a^ *Mann-Whitney test*

**Fig. 1:**
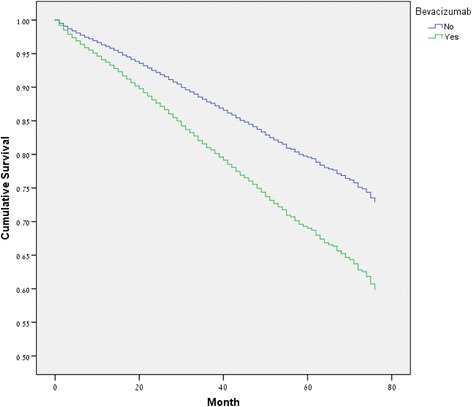
Cumulative Survival. Cumulative survival is greater in patients who were not exposed to bevacizumab

During follow-up (maximal follow-up of 73 months), 1063 (19.7%) patients who used bevacizumab died, compared to 1298 (12.1%) in the control group (*P* < .001). Cumulative survival was greater in the control group (Table 2, Fig. 1). After adjusted Cox regression, mortality was greater for the treatment group, OR = 1.69, (95% C.I. 1.54–1.84), *P* < .001(Table 3).

**Table 2:** Survival (Life Table)

Beva-cizumab	Year	Entering Interval	Withdrawing during Interval	Exposed to Risk	Terminal Events	Proportion Terminating	Cumulative Proportion Surviving at End of Interval
No	0–1	10,756	3050	9231	517	.06	.94
	1–2	7189	2336	6021	327	.05	.89
	2–3	4526	1443	3805	219	.06	.84
	3–4	2864	963	2383	124	.05	.80
	4–5	1777	770	1392	73	.05	.76
	5–6	934	611	629	32	.05	.72
	6–7	291	285	149	6	.04	.69
Yes	0–1	5385	1527	4622	470	.10	.90
	1–2	3388	1099	2839	238	.08	.82
	2–3	2051	631	1736	168	.10	.74
	3–4	1252	407	1049	99	.09	.67
	4–5	746	313	590	63	.11	.60
	5–6	370	249	246	21	.09	.55
	6–7	100	96	52	4	.08	.51

**Table 3:** Mortality (Cox regression)

Variables	B	S.E.	Wald (df = 1)	p-value	OR
Gender (male)	.053	.043	1.507	.220	1.054
Age start (Year)	.013	.004	12.927	.000	1.013
Socioeconomic (low)	.083	.057	2.093	.148	1.086
Smoking	.162	.061	7.135	.008	1.176
Alcohol	.323	.240	1.809	.179	1.381
Hypertension	.032	.056	.322	.570	1.032
Diabetes	.015	.046	.106	.745	1.015
Obesity	.071	.054	1.724	.189	1.073
Congestive heart failure	.172	.055	9.779	.002	1.187
Liver cancer	.775	.516	2.255	.133	2.170
Ischemic heart disease	.000	.046	.000	.993	1.000
Cerebrovascular accident	.153	.051	9.065	.003	1.166
Bevacizumab use	.527	.043	153.744	.000	1.694

The mean number of injections was significantly lower in patients who died 6.1 (6.43) vs. survived 8.3 (8.82) years, *t*(2158) = 8.98, *P* < .001. Patients died after having being treated during 10.50 ± 13.57 months, 18.8 ± 16.80 months after the last injection.

## Discussion

We report increased mortality in patients treated with bevacizumab for wet AMD, compared to age and gender matched individuals for whom there was no record of a prescription to any anti-VEGF agent.

Bevacizumab is known to escape the eye, reach the general circulation, and inhibit systemic VEGF-A [3]. VEGF-A is involved in homeostasis and healing in many systems. As an anti-angiogenic agent, bevacizumab may impair the ability of vascular tissues to contribute to healing [4, 22]. Impairment of normal healing rather than direct injury to vital organs seems to explain the long-term adverse effects observed for bevacizumab. However, this is difficult to detect in patients who receive intravenous doses of bevacizumab, due to the reduced survival inherent to the malignant condition being treated; nevertheless, apprehensions have been raised [23].

In the current study, risk factors previously reported to be common to AMD and cardiovascular disease [24–26] were found to account in part for the increased mortality of the bevacizumab users. Nevertheless, the increased mortality persisted after adjusting for cardiovascular risk factors. Mortality specifically associated with wet AMD has been attributed to the visual impairment it induces [27]. Anti-VEGF treatments have been demonstrated to restore vision [2, 5, 7–13, 28, 29]. Hence, our results support the contribution of bevacizumab to increased mortality, beyond the condition of wet AMD.

It must be noted that, in our study, cases and controls differ on just about every risk factor for death (Table 1). If inclusion of categorical and quantitative variables did not fully capture the association between the factors and death, then there may be residual confounding. A control group that would circumvent those methodologic issues would consist of patients with wet AMD who did not receive injections. In our era, this population does not exist. Comparing two populations with wet AMD in different periods would introduce other serious bias. There would be two major flaws if we wanted to compare AMD patients without neovascularization with patients having the neovascular form. First, information extracted from such electronic medical records lack the precision required to be certain that patients registred as having dry AMD do not suffer from the neovascular form in at least one of their eyes. Then, since wet and dry AMD do not necessarily share the same risk profile [26, 30, 31], the risk of confounding by indication would persist.

A limitation of this study is that our database does not differentiate between unilateral and bilateral injections, and provides only limited information on ocular conditions. Visual acuity, for instance, is not recorded. Patients who went on to use other anti-VEGF treatments were excluded from this study. This might introduce a bias into the comparison death rates, as indivuals receiving second line treatments do not necessarily share the same risk profile as people responding to bevacizumab. However, to our knowledge, such a difference has never been reported.

Another weakness of our data is that the cause of death is not available. Nevertheless, all-cause mortality has some advantages as a principle end point, given the potential for misclassifying the cause of death [32].

The strength of this study is the inclusion of a large number of patients who received bevacizumab and no other anti-VEGF therapy for wet AMD, with detailed registration of comorbidities and socioeconomic data, which enabled suitable matching and multivariate analysis.

## Conclusions

The findings presented raise questions regarding the use of bevacizumab for wet AMD. Other anti-VEGF intraocular compounds are used as second-line therapy in Israel. Due to the observed delay between the last bevacizumab injection and death, our data do not enable valid assessment of the effects of ranibizumab and aflibercept on mortality. Additional data is needed to corroborate our worrying observation that bevacizumab intraocular injections may be associated with increased mortality. If confounding by indication could be ruled out but economic reasons precluded immediate interruption of bevacizumab therapy for wet AMD, it would be crucial to define groups of higher and lower risk, to enable physicians and patients to discuss the systemic impact of ocular therapy and adequately balance expected gains and risks.

## Abbreviations

AMD: Age-related macular degeneration
FDA: Food and drug administration
VEGF: vascular endothelial growth factor
